# An explainable ensemble machine learning model using baseline blood transcriptomics to predict Parkinson's disease motor progression

**DOI:** 10.3389/fdgth.2026.1774436

**Published:** 2026-02-18

**Authors:** Yelda Fırat

**Affiliations:** Department of Computer Engineering, Mudanya University, Bursa, Türkiye

**Keywords:** mitochondrial dysfunction, Parkinson's disease, PPMI, RNA-seq, SHAP

## Abstract

**Introduction:**

Predicting Parkinson's disease (PD) motor progression remains challenging despite advances in neuroimaging. Blood-based transcriptomic profiling offers a more accessible and cost-effective alternative. This study aimed to develop and validate a machine learning approach using blood-based transcriptomic data to predict 12-month motor severity in PD and to identify the transcriptomic features and biological pathways most strongly associated with progression.

**Methods:**

A Stacking Regressor ensemble model combining three gradient boosting algorithms (XGBoost, LightGBM, CatBoost) was developed using baseline Parkinson's Progression Markers Initiative (PPMI) data (*n* = 390), integrating blood RNA sequencing (RNA-seq) and clinical features to predict 12-month UPDRS Part III scores. SHapley Additive exPlanations (SHAP) analysis was applied to identify key prognostic features, evaluating seven PD risk genes (SNCA, LRRK2, GBA, PRKN, PINK1, PARK7, VPS35) and pathway scores for mitochondrial dysfunction, neuroinflammation, and autophagy.

**Results:**

On an independent test set (*n* = 78), the model achieved a Coefficient of Determination (R²) of 0.551 and Mean Absolute Error (MAE) of 6.01. SHAP analysis identified the baseline UPDRS × PINK1 interaction (UPDRS_BL × PINK1) as the most influential feature (mean |SHAP| = 0.283). Among transcriptomic features, VPS35 (mean |SHAP| = 0.010), GBA, and LRRK2 were most prominent. Mitochondrial dysfunction showed the highest pathway contribution (mean |SHAP| = 0.008).

**Discussion:**

The study establishes that machine learning integrating blood transcriptomics and clinical data effectively predicts motor progression in PD. Crucially, the interplay between initial clinical state and specific genetic backgrounds-particularly PINK1-is a more powerful prognostic indicator than any factor alone. This study provides systematic evidence that mitochondrial dysfunction is a dominant prognostic signal for disease progression, nominating key genes and pathways for future mechanistic and therapeutic investigation.

## 1 Introduction

PD is a neurodegenerative disease characterized by progressive dopaminergic neuron loss, affecting approximately 1% of individuals over 60 worldwide. Clinical course varies greatly; some patients remain stable for years, while others experience rapid motor and cognitive decline. This heterogeneity underscores the need for reliable biomarkers predicting early progression. Accurate progression prediction is critical for personalized treatment strategies and optimizing patient selection in clinical trials.

In recent years, artificial intelligence (AI) methods have emerged as powerful tools for predicting PD progression and understanding underlying biological mechanisms. PD's clinical and genetic heterogeneity make prognosis difficult with traditional methods, whereas AI models effectively process complex data structures. AI models increasingly succeed at diagnosing and monitoring PD (1). Various data types predict PD progression: motor activity from wearable sensors (2), surface electromyography signals (3), and force platform balance data (4) successfully model motor symptom changes. Transcriptomic data, particularly blood-based RNA-seq, offer rich prognostic markers by revealing immune cell and gene expression changes in early disease stages (5). Deep recurrent neural networks trained on RNA-seq data accurately predict PD progression (6). Deep sequencing of small non-coding RNAs (sncRNAs) reveals regulatory modules and discriminative transcriptomic features during progression (7). These approaches critically identify patient subtypes and develop personalized progression models (8).

However, the black-box mechanisms of complex AI models remain a major obstacle to clinical integration. Explainable Artificial Intelligence (XAI) methods, such as SHapley Additive exPlanations (SHAP), address this by revealing which features influence predictions and how. SHAP analysis enhances model interpretability and reliability across applications, from drug development (9) to hospital data (10) and chronic disease management (11). XAI's potential and challenges in healthcare remain active areas of research (12, 13).

The machine learning model used in this study employs ensemble learning, combining multiple learners for robust, accurate predictions. Ensemble learning effectively predicts Parkinson's disease progression (8, 14). Ensemble methods outperform individual models, particularly in observational, noisy healthcare datasets (15, 16). XGBoost is widely used in medical prediction models due to its high performance and natural compatibility with XAI techniques like SHAP (17).

In this study, an integrated approach is presented combining machine learning with molecular genetic analysis. Using baseline blood RNA-seq and clinical data from the Parkinson's Progression Markers Initiative (PPMI) dataset, a Stacking Regressor model was developed to predict 12-month motor status (UPDRS Part III score). The model generates robust predictions by combining three gradient boosting variants (XGBoost, LightGBM, CatBoost). Interpretability was ensured through SHAP analysis, identifying features most important for predicting progression. SHAP analysis evaluated seven PD risk genes (SNCA, LRRK2, GBA, PRKN, PINK1, PARK7, VPS35). VPS35 showed the highest individual prognostic importance (SHAP = 0.010), followed by GBA and LRRK2. However, baseline UPDRS × PINK1 interaction (UPDRS_BL × PINK1) exhibited the highest prognostic value among all features (SHAP = 0.283). PINK1 and PARK7 have been shown to regulate mitophagy, clearing damaged mitochondria via autophagy (18, 19). In the PINK1/PARKIN pathway, PINK1 marks damaged mitochondria and activates PARKIN (20). PARK7 (DJ-1) provides cellular protection against oxidative stress and regulates mitochondrial homeostasis (21). Although SNCA (α-synuclein) is the best-known genetic and pathological marker of Parkinson's disease, its blood-based expression showed little prognostic significance. Among biological pathways, mitochondrial dysfunction exhibited the highest SHAP value (0.008), revealing that baseline motor severity, gene-clinical interactions (UPDRS_BL × PINK1), PD risk genes (VPS35, GBA, LRRK2), and mitochondrial dysfunction most contributed to predictions. By evaluating individual and interactive contributions of these seven PD risk genes, this study demonstrates the strong association of mitochondrial dysfunction with disease progression. This integrated approach provides predictive power while illuminating underlying molecular mechanisms, advancing personalized medicine and targeted therapy development.

## 2 Method

### 2.1 PPMI dataset and data preprocessing

Data were obtained from the Parkinson's Progression Markers Initiative (PPMI) cohort, supported by the Michael J. Fox Foundation (22). PPMI is a multicenter, observational study launched in 2010 to monitor PD progression and discover biomarkers. All data were downloaded in accordance with ethical approval and data use agreements.

The PPMI dataset comprises genomic data (RNA-seq) and clinical data. Genomic data include RNA sequencing from patients’ blood samples, with gene expression normalized to Transcripts Per Million (TPM) units to ensure fair comparison between samples and genes. Clinical data include patients' demographic information, diagnoses, and motor assessment scores. UPDRS Part III scores, which measure motor function, serve as the model's target variable.

Data preprocessing was performed in three main steps. First, UPDRS Part III scores from Baseline (BL) and Visit 04 (V04, 12-month) were processed. The 12-month UPDRS Part III score (UPDRS_V04) was used as the continuous target variable. A classification variable (Progressor_Type) was created based on 12-month UPDRS change (*Δ*UPDRS = UPDRS_V04−UPDRS_BL), with patients classified as fast progressors if *Δ*UPDRS ≥ 5. This 5-point threshold was selected based on established literature defining the minimal clinically important difference (MCID) for UPDRS Part III motor scores. Shulman et al. (23) identified 5.2 points as the moderate clinically important difference, and subsequent studies have consistently used 5 points as the threshold for clinically meaningful motor progression (24, 25). This variable was used for stratified sampling to ensure balanced representation of fast and slow progressors in training and test sets.

Second, the PPMI Project 133 RNA-sequencing data were processed. Baseline RNA-seq data were filtered to exclude low-quality samples and genes with low expression (TPM < 1 in 90% of samples), reducing noise and improving model performance. A single-time-point (baseline-only) approach was adopted to predict 12-month progression from a single blood sample at diagnosis, rather than a longitudinal design (8, 16). This approach enables clinical applicability by determining progression risk from a single baseline blood sample without requiring repeated patient visits.

To ensure methodological rigor and prevent label leakage, all predictor variables were measured at baseline (Visit 0, t = 0), while the target variable was measured at a future time point (Visit 04, t = 12 months). Specifically, baseline UPDRS Part III score (UPDRS_BL), age, gender, baseline blood RNA-seq gene expression profiles, PD risk gene expression levels, biological pathway scores, and interaction terms (e.g., UPDRS_BL × PINK1) were all measured at t = 0. The target variable, 12-month UPDRS Part III score (UPDRS_V04), was measured at t = 12. This temporal separation ensures that all predictors are measured before the outcome, consistent with standard prognostic modeling practices in Parkinson's disease research (6, 8, 16). Using baseline disease severity (UPDRS_BL) to predict future disease severity (UPDRS_V04) is clinically valid and does not constitute label leakage, as these represent measurements of the same clinical construct at different time points. The variable DELTA_UPDRS (UPDRS_V04 - UPDRS_BL), which quantifies 12-month motor progression, was used only for stratified sampling to ensure balanced representation of fast and slow progressors in training and test sets, but was not used as a predictor variable in the model.

Third, clinical and RNA-seq data were merged using patient identifiers (PATNO), and patients with missing data were excluded. Feature engineering was then applied. The top 100 genes most correlated with 12-month UPDRS change (*Δ*UPDRS) were selected from approximately 20,000 genes remaining after low-expression filtering. Pearson correlation coefficients were calculated between each gene's baseline expression and *Δ*UPDRS, and genes were ranked by absolute correlation magnitude. The top 100 genes (correlation range: |r| = 0.45 to 0.15) were selected to form the most important transcriptomic features. This correlation-based selection reduces dimensionality while retaining genes with the strongest linear associations with motor progression. Additionally, baseline expression levels of seven PD risk genes (SNCA, LRRK2, GBA, PRKN, PINK1, PARK7, VPS35) were added as separate features (26, 27). Pathway scores were calculated as the mean expression of gene sets associated with neuroinflammation, mitochondrial dysfunction, and autophagy to assess the contribution of biological mechanisms to progression. Three interaction features were established based on pre-specified biological hypotheses regarding the PINK1/PARKIN mitophagy pathway (19, 21), rather than data-driven discovery: PINK1 × PARK7 (mitochondrial quality control interaction), AGE × PINK1 (age-dependent mitochondrial effects), and UPDRS_BL × PINK1 (baseline severity modulation of PINK1 prognostic value).

Outliers in UPDRS_V04 scores were identified using the Interquartile Range (IQR) method, and 2 patients were excluded to reduce the impact of extreme values on model performance. The final dataset contains 390 patients and 116 features: 3 clinical covariates (UPDRS_BL, AGE, GENDER), 100 most correlated genes, 7 PD risk genes, 3 pathway scores, and 3 interaction features. UPDRS_V04 (continuous variable) was used as the target variable.

Before model training, all continuous features (gene expression and clinical covariates) were standardized using StandardScaler (mean = 0, standard deviation = 1) to prevent features on different scales from adversely affecting performance. A Power Transformation (Yeo-Johnson method) was applied to the target variable (UPDRS_V04) to normalize its distribution, enhancing the model's learning capacity and predictive performance (28). Model predictions were inverse-transformed to the original scale.

The dataset was divided into a training/validation set (80%, *n* = 312) and an independent clinical test set (20%, *n* = 78) using stratified sampling to preserve the balance between progressor and non-progressor classes (Fast vs. Slow). Hyperparameter optimization was performed using Bayesian optimization (Optuna) with 7-fold cross-validation on the training/validation sets (*n* = 312). Optuna's Tree-structured Parzen Estimator (TPE) algorithm systematically explored the hyperparameter space across 30 trials to maximize mean cross-validation R^2^.

For each base model, the following hyperparameter ranges were explored: XGBoost (n_estimators: 100–250, max_depth: 3–7, learning_rate: 0.01–0.1, subsample: 0.6–0.9, colsample_bytree: 0.6–0.9, min_child_weight: 1–8, L1 regularization: 0.01–1.0, L2 regularization: 0.1–5.0); LightGBM (n_estimators: 100–250, max_depth: 3–7, learning_rate: 0.01–0.1, subsample: 0.6–0.9, colsample_bytree: 0.6–0.9, min_data_in_leaf: 5–25, L1 regularization: 0.01–1.0, L2 regularization: 0.1–5.0); CatBoost (iterations: 100–250, depth: 3–7, learning_rate: 0.01–0.1, subsample: 0.6–0.9, L2 regularization: 0.1–5.0). For the meta-learner (Huber Regressor), alpha (regularization strength: 0.01–1.0) and epsilon (robustness parameter: 1.0–2.0) were optimized. Learning rate and regularization parameters were sampled on a logarithmic scale to efficiently explore multiple orders of magnitude. After optimization, the final model with optimal hyperparameters was retrained on the entire training/validation set (*n* = 312) and evaluated using 7-fold cross-validation and the independent clinical test set (*n* = 78). The independent test set was never used for hyperparameter optimization or training and was reserved for assessing real-world performance.

All model development, evaluation, and visualization were carried out using Python 3.11, Scikit-learn, and Matplotlib. The code, trained model, curated analysis dataset, and aggregate results (feature importance and SHAP values) that support the findings of this study are openly available on [GitHub] at [https://github.com/yeldafrt/PD-Blood-Transcriptomics-SHAP]. The repository includes frozen environment files (requirements.txt with exact package versions), Docker container configuration (Dockerfile), and Conda environment specification (environment.yml) to ensure computational reproducibility.

### 2.2 Modeling and performance evaluation

In this study, a Stacking Regressor model was used to predict 12-month motor state (UPDRS_V04) from baseline data. Stacking is an ensemble learning technique that combines multiple machine learning models to achieve robust predictions (29). Three gradient boosting variants (XGBoost, LightGBM, CatBoost) served as base models, with their predictions combined by a Huber Regressor meta-model. Gradient boosting models capture complex linear and nonlinear relationships across heterogeneous data, including clinical features (e.g., UPDRS scores, age, gender) and genomic features (e.g., gene expression profiles). Each variant's distinct optimization strategies and regularization techniques increase ensemble diversity and reduce the risk of overfitting.

The model was evaluated using a two-stage strategy. First, 7-fold cross-validation was applied to the training/validation set (*n* = 312), dividing the dataset into 7 equal parts, with each serving as the validation set in turn to assess learning capacity and internal consistency. Cross-validation reduces the risk of overfitting and provides reliable performance estimates. Second, the model was evaluated on an independent clinical holdout set (*n* = 78) that was never used for hyperparameter optimization or training, to measure real-world performance and generalization to new patients. This two-stage strategy comprehensively tests both learning capacity and generalization.

Model performance was measured using three commonly used metrics for regression problems: R^2^ (the proportion of variance explained by the model), MAE, and Root Mean Squared Error (RMSE). The R^2^ value indicates how much of the variance in the target variable the model explains, ranging from 0 to 1; values closer to 1 indicate better performance. MAE expresses the average deviation of predictions from the actual values in UPDRS score units, with lower values indicating better performance. RMSE is a metric that penalizes larger errors more heavily, and lower values are also preferred. Table 1 summarizes the model's performance metrics on 7-fold cross-validation and an independent clinical test set.

**Table 1:** Model performance metrics.

Evaluation set	R^2^	MAE	RMSE
7-Fold CV			
Fold 1	0.574	6.04	7.40
Fold 2	0.480	6.56	8.45
Fold 3	0.498	5.91	7.54
Fold 4	0.571	6.21	7.38
Fold 5	0.506	6.30	8.55
Fold 6	0.428	6.22	8.38
Fold 7	0.537	5.81	7.30
Mean ± SD (*n* = 312)	0.513 ± 0.052	6.15 ± 0.25	7.86 ± 0.57
Clinical holdout (*n* = 78)	**0**.**551**	**6**.**01**	**7**.**21**

Bold values indicate the best performance achieved across all evaluation sets.

Table 1 summarizes the model's performance metrics from 7-fold cross-validation and an independent clinical test set. The 7-fold cross-validation results (*n* = 312) demonstrate consistent performance, with R^2^ ranging from 0.428 to 0.574 (mean: 0.513 ± 0.052). This low standard deviation indicates stable performance across patient subgroups and acceptable fold variation. MAE ranges from 5.81 to 6.56 points (mean: 6.15 ± 0.25), showing that predictions deviate from actual values by approximately 6 points on average, which is clinically acceptable relative to the UPDRS Part III significance threshold (5 points).

Results from an independent clinical holdout set (*n* = 78) demonstrate strong generalization ability. This set, never used for hyperparameter optimization or training, was reserved for evaluating real-world performance. The R^2^ (0.551) exceeded the cross-validation average (0.513), indicating better performance on new patients without overfitting. MAE (6.01 points) was lower than the cross-validation score (6.15 points), confirming the reliability of clinical predictions. RMSE (7.21) indicates rare large errors. The consistency between cross-validation and holdout results (R^2^: 0.513 vs. 0.551) demonstrates robust model structure and reliable performance across patient populations.

### 2.3 SHAP analysis and model interpretability

For clinical acceptance, machine learning models must achieve high predictive performance and have explainable decision-making mechanisms. SHAP analysis was applied to improve model interpretability and quantitatively assess each feature's contribution to progression predictions. SHAP, based on game theory Shapley values, makes black-box model decisions explainable (30).

SHAP analysis was performed on an independent clinical test set (*n* = 78) for three feature categories: (1) Clinical Features – baseline UPDRS Part III, age, sex, and their interactions with PD risk genes (e.g., UPDRS_BL × PINK1); (2) PD Risk Genes – individual expression levels of seven literature-supported risk genes (SNCA, LRRK2, GBA, PRKN, PINK1, PARK7, VPS35) (26, 27) and (3) Pathway Scores – pathway scores representing biological processes (mitochondrial dysfunction, neuroinflammation, autophagy). SHAP values were calculated separately for each base model (XGBoost, LightGBM, CatBoost) using TreeExplainer and then averaged across all three estimators to produce ensemble-averaged SHAP values. This approach ensures that feature importance reflects the collective contribution across all models in the stacking ensemble.

## 3 Results

**Figure 1:**
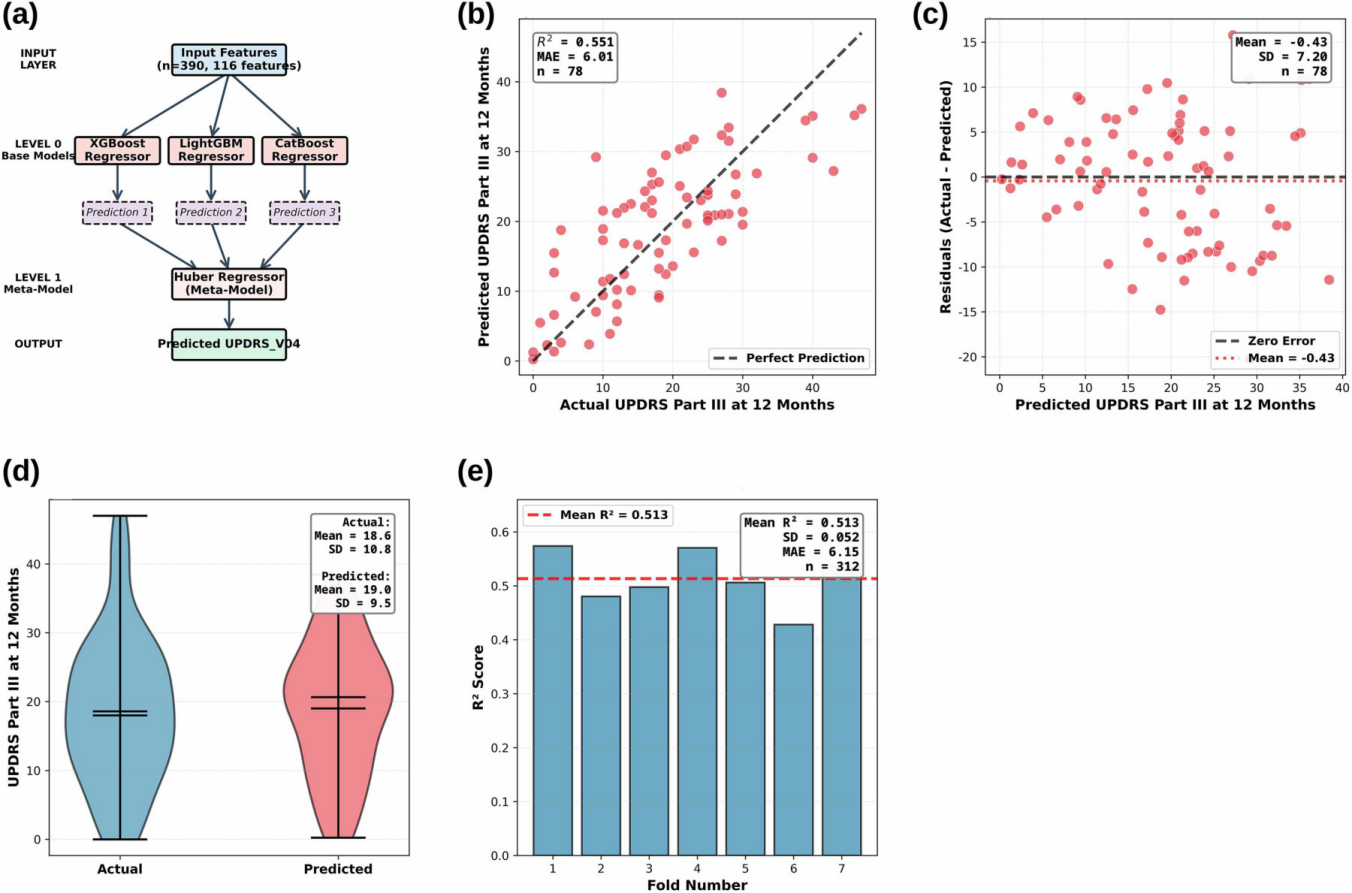
Stacking regressor model architecture and comprehensive performance evaluation. **(a)** model architecture; **(b)** Predicted vs. actual (*n* = 78); **(c)** Residuals; **(d)** Distribution comparison; **(e)** 7-fold CV bar chart (*n* = 312).

A Stacking Regressor ensemble model integrating baseline clinical and blood-based transcriptomic data was developed to predict 12-month motor progression in Parkinson's disease. The model achieved R^2^ = 0.551 and MAE = 6.01 on an independent clinical test set (*n* = 78), demonstrating strong predictive performance using only baseline blood RNA-seq data. The stacking ensemble model architecture and comprehensive performance evaluation results across multiple visualization approaches are presented in Figure 1.

Figure 1a shows the two-level stacking ensemble architecture. Level 0 comprises three gradient-boosting base models (XGBoost, LightGBM, CatBoost) trained on 390 patients with 116 features (100 genes + 16 clinical/pathway features). Level 1 uses a Huber Regressor meta-model to combine base model predictions through optimal linear weighting, producing the final UPDRS_V04 prediction while maintaining robustness against outliers.

Figure 1b visualizes model performance on the independent clinical validation set. The scatter plot shows actual vs. predicted UPDRS Part III scores at 12 months, with the dashed line representing perfect prediction (y = x). The model achieved R^2^ = 0.551 and MAE = 6.01 on patients not used during training, demonstrating strong generalization. The balanced distribution of points around the perfect-prediction line indicates no systematic bias.

Figure 1c presents a residual plot to evaluate the error structure and prediction consistency. The plot shows residuals (actual - predicted) vs. predicted UPDRS Part III scores, with the dashed line representing zero error and the dotted line indicating the mean residual (Mean = −0.43). The random distribution of residuals around zero suggests no systematic bias. The residual standard deviation (SD = 7.20) is consistent with RMSE (7.21). The absence of heteroscedasticity indicates consistent performance across the prediction range.

Figure 1d compares actual and predicted UPDRS Part III score distributions using violin plots. The actual scores (Mean = 18.6, SD = 10.8) and predicted scores (Mean = 19.0, SD = 9.5) exhibit similar distributions, indicating that the model successfully captured the true data distribution. The slightly narrower predicted distribution (SD: 9.5 vs. 10.8) suggests regression to the mean. The minimal difference between the mean values (18.6 vs. 19.0) confirms the absence of systematic bias and balanced predictions.

Figure 1e shows 7-fold cross-validation results on the training/validation set (*n* = 312) using a bar chart to display fold-by-fold R^2^ scores. The model achieved R^2^ = 0.513 ± 0.052 and MAE=6.15 ± 0.25, demonstrating consistent performance across patient subgroups with R^2^ values ranging from 0.428 to 0.574 across the seven folds.

**Figure 2:**
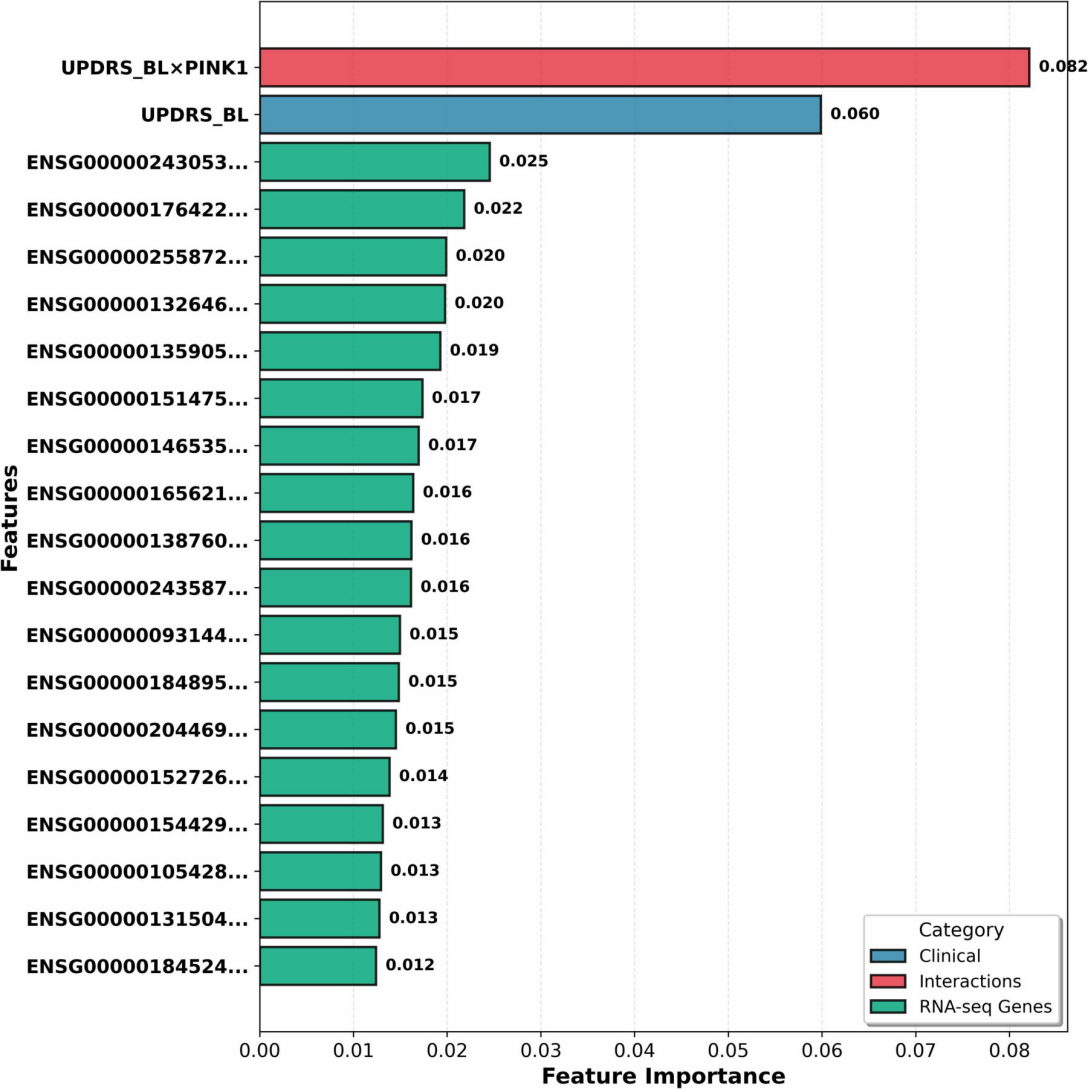
Top 20 features contributing to stacking regressor model predictions.

Figure 2 presents the 20 features that most contribute to the model's predictive performance, grouped into three main categories.

The feature importance values in Figure 2 were calculated by averaging across three algorithms (XGBoost, LightGBM, and CatBoost). The most important feature was the UPDRS_BL × PINK1 interaction (0.082), followed by baseline UPDRS score (0.060). Notably, 90% (18/20) of top features are RNA-seq gene expression data, indicating that transcriptomic data play a critical role in predicting Parkinson's disease progression. Clinical features (blue), gene-gene interactions (red), and RNA-seq genes (green) are color-coded, with normalized importance scores shown on the right.

**Figure 3:**
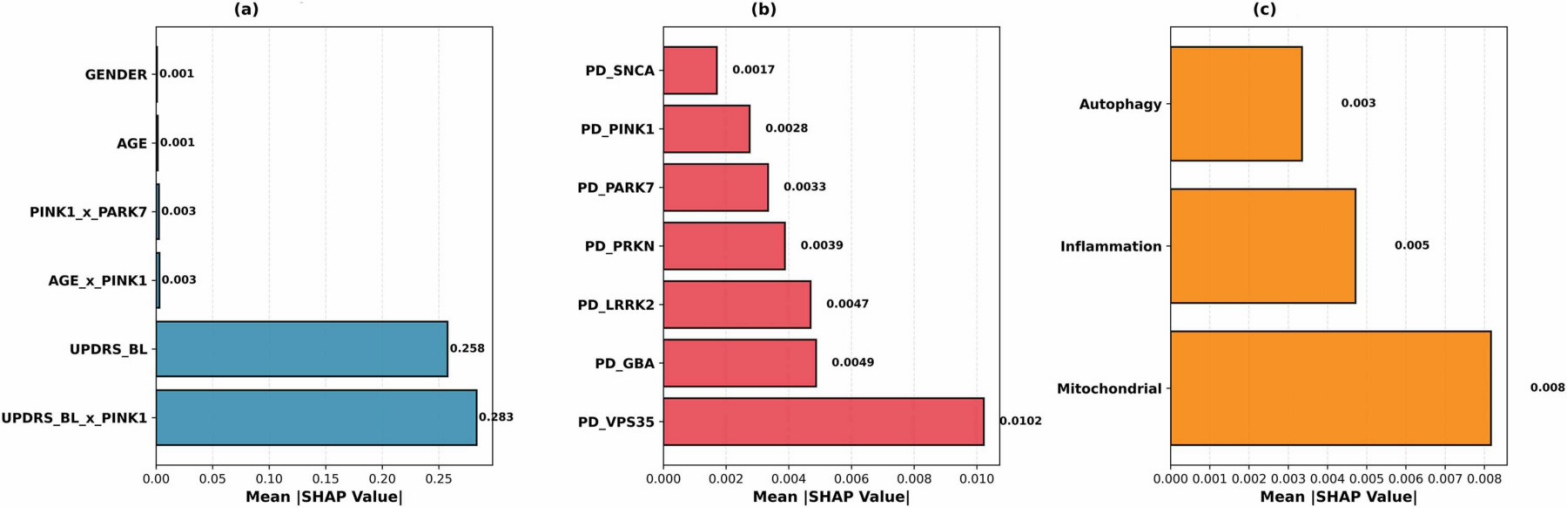
SHAP analysis for model interpretability. **(a)** prediction contributions of clinical features; **(b)** PD risk genes; **(c)** Pathway scores.

Figure 3 visualizes SHAP results in three panels.

In Figure 3, Panel (a) shows that the UPDRS_BL × PINK1 interaction has the highest SHAP value (0.283), followed by the baseline UPDRS score (0.258). Demographic features (age, sex) show minimal contribution (<0.002). Panel (b) shows VPS35 has the highest SHAP value among seven PD risk genes (0.010), followed by GBA (0.005) and LRRK2 (0.005). The low individual SHAP values for PINK1 and SNCA suggest they contribute to predictions primarily through interactions. Panel (c) shows that the mitochondrial dysfunction pathway score has the highest SHAP value (0.008), followed by neuroinflammation (0.005) and autophagy (0.003), supporting mitochondrial dysfunction as a dominant prognostic signal in PD progression.

## 4 Discussion

Model performance is competitive with similar cross-sectional approaches. While Nguyen et al. (31) achieved R^2^ = 0.558 using neuroimaging, the proposed ensemble model achieved R^2^ = 0.551 using baseline blood RNA-seq data alone, offering key advantages: (1) explainability through SHAP analysis revealed gene-clinical interactions (e.g., UPDRS_BL × PINK1) as critical predictive features of progression, addressing not only predictive accuracy but also the features underlying the predictions; (2) blood-based transcriptomic profiling provides a minimally invasive, accessible alternative to expensive brain imaging; and (3) the proposed single-timepoint baseline approach enables immediate prognostic assessment from a single blood sample at diagnosis, without requiring longitudinal data or repeated visits. Although longitudinal designs achieve higher performance using prior-visit data (R^2^≈0.69) (6), the proposed cross-sectional baseline design offers superior clinical applicability: newly diagnosed patients can receive immediate predictions of progression risk, facilitating early intervention planning and patient stratification in clinical trials.

Recent studies demonstrate the effectiveness of ensemble learning for predicting Parkinson's progression (8, 14). This study extends this paradigm in two ways: First, unlike Dadu et al.'s (2022) categorical classification, the proposed Stacking Regressor predicts 12-month UPDRS scores as continuous values, enabling more precise individualized forecasts. Second, SHAP analysis (30) quantitatively explains which features (UPDRS_BL × PINK1, VPS35, mitochondrial pathway) predict progression, adding interpretability to the ensemble approach.

The model's success derives from the synergistic integration of clinical features and gene expression profiles. SHAP analysis revealed biologically meaningful, interpretable features, with clinical and interaction features dominating predictions. The two highest contributors were the interaction between baseline UPDRS and PINK1 expression (UPDRS_BL × PINK1, SHAP = 0.283) and baseline UPDRS itself (SHAP = 0.258), indicating that initial disease severity is strongly associated with progression rate and this association varies with genetic factors like PINK1. Among seven Parkinson's risk genes (27), VPS35, which is involved in endosomal trafficking and lysosomal function, had the highest SHAP value (0.010), followed by the classical risk genes GBA (0.005) and LRRK2 (0.005). Notably, PINK1 and SNCA showed relatively low individual SHAP values, suggesting their effects emerge primarily through interactions (e.g., UPDRS_BL × PINK1) or that blood-based RNA-seq incompletely captures their brain-specific roles. Among biological pathways, mitochondrial dysfunction exhibited the highest SHAP value (0.008), followed by neuroinflammation (0.005) and autophagy (0.003). This is consistent with mitochondrial dysfunction's strong association with Parkinson's progression, given dopaminergic neurons' high energy demands and their susceptibility to oxidative stress (18, 19, 21).

Demographic features (age, sex) contributed minimally to the model's predictions (mean |SHAP| < 0.002). Specifically, the negligible contribution of sex is consistent with the lack of a significant difference in 12-month motor progression between males and females in this cohort (*p* = 0.54). The dataset shows a male-predominant sex distribution (1.42:1 male-to-female ratio), which reflects the known epidemiology of Parkinson's disease. Similarly, age showed minimal predictive contribution despite being a known risk factor for Parkinson's disease; this may be because baseline motor severity (UPDRS_BL) already captures age-related disease burden. The model's predictive power is primarily driven by baseline clinical severity and transcriptomic features rather than demographic variables.

This study offers notable strengths. Integrating machine learning with SHAP analysis provides moderate prediction accuracy while ensuring model transparency. Clinically, the baseline blood RNA-seq approach offers a minimally invasive, cost-effective alternative to neuroimaging for progression risk assessment. Additionally, sharing the ensemble model and analysis code as open source ensures reproducibility.

This study has limitations. First, this study is limited to the PPMI cohort. While the model was rigorously validated using an independent holdout test set (*n* = 78, 20%) that was completely withheld from training and hyperparameter optimization, this represents internal validation within the same cohort rather than external validation in an entirely independent dataset. Although this approach demonstrates robust generalization to unseen patients within the PPMI population, external validation is essential to confirm generalizability across diverse populations that may differ in demographic characteristics (age, ethnicity, geographic distribution), disease phenotypes (severity, subtypes, progression patterns), RNA-seq protocols (sequencing platforms, batch effects), and clinical assessment methods (rater variability, protocol differences). Validation in independent cohorts from different centers and populations is required to establish clinical utility and real-world applicability. Second, the model predicts 12-month progression; validation for longer-term forecasts is needed. Third, blood-based RNA-seq may not fully reflect brain-specific pathology, potentially explaining low SHAP values for genes such as SNCA. Fourth, the model focuses exclusively on motor symptoms (UPDRS Part III) rather than non-motor features. Fifth, treatment changes during the 12-month follow-up period were not modeled. Changes in medication (e.g., levodopa dosage adjustments) can significantly influence UPDRS scores, acting as a major confounding variable. This limitation means the model predicts progression based on the combined effects of natural disease progression and treatment, rather than isolating the biological progression alone. Future studies should incorporate time-varying medication data as a covariate to disentangle these effects. Finally, this study does not include environmental exposure variables (e.g., pesticide exposure, air pollution, occupational exposures) or site/center identifiers that could account for geographic variation in environmental risk factors. Environmental factors may influence disease progression and interact with genetic risk factors. Future studies integrating blood transcriptomics with environmental exposure data could provide a more comprehensive understanding of gene-environment interactions in Parkinson's disease progression.

Future studies should integrate blood RNA-seq with brain imaging, cerebrospinal fluid biomarkers, and genetic data to develop more comprehensive prognostic models. Investigating non-coding regulatory elements (promoters, enhancers, miRNAs) and epigenetic modifications could reveal deeper mechanisms of progression. Therapeutically, mitochondrial biomarkers could predict treatment response, warranting randomized trials of mitochondria-targeted therapies (coenzyme Q10, creatine). Clinically, SHAP-based patient-specific progression profiles could inform personalized treatment strategies. Finally, validating the ensemble model in independent multi-center cohorts will be critical for clinical integration.

## 5 Conclusion

This study establishes that machine learning integrating blood transcriptomics and clinical data effectively predicts motor progression in Parkinson's disease. Crucially, the interplay between initial clinical state and specific genetic backgrounds—particularly PINK1—is a more powerful prognostic indicator than any factor alone. This study provides systematic evidence that mitochondrial dysfunction is a dominant prognostic signal for disease progression, highlighting key genes and biological pathways as promising targets for future mechanistic and therapeutic investigation.

## Data Availability

The original contributions presented in the study are included in the article/Supplementary Material, further inquiries can be directed to the corresponding author.
